# Majority Rules with Random Tie-Breaking in Boolean Gene Regulatory Networks

**DOI:** 10.1371/journal.pone.0069626

**Published:** 2013-07-26

**Authors:** Claudine Chaouiya, Ouerdia Ourrad, Ricardo Lima

**Affiliations:** 1 Instituto Gulbenkian de Ciência, Oeiras, Portugal; 2 Theoretical Physics Laboratory, A. Mira University of Bejaia, Bejaia, Algeria; 3 Dream & Science Factory, Marseille, France; 4 Institute for Complexity Sciences, Lisbon, Portugal; Universidad de Zarazoga, Spain

## Abstract

We consider threshold Boolean gene regulatory networks, where the update function of each gene is described as a majority rule evaluated among the regulators of that gene: it is turned ON when the sum of its regulator contributions is positive (activators contribute positively whereas repressors contribute negatively) and turned OFF when this sum is negative. In case of a tie (when contributions cancel each other out), it is often assumed that the gene keeps it current state. This framework has been successfully used to model cell cycle control in yeast. Moreover, several studies consider stochastic extensions to assess the robustness of such a model.

Here, we introduce a novel, natural stochastic extension of the majority rule. It consists in randomly choosing the next value of a gene only in case of a tie. Hence, the resulting model includes deterministic and probabilistic updates. We present variants of the majority rule, including alternate treatments of the tie situation. Impact of these variants on the corresponding dynamical behaviours is discussed. After a thorough study of a class of two-node networks, we illustrate the interest of our stochastic extension using a published cell cycle model. In particular, we demonstrate that steady state analysis can be rigorously performed and can lead to effective predictions; these relate for example to the identification of interactions whose addition would ensure that a specific state is absorbing.

## Introduction

Cellular processes are driven by large and heterogeneous interaction networks that are being uncovered thanks to tremendous technological advances. In this context, a range of modelling frameworks has been deployed to represent and analyse biological networks, aiming at better understanding these complex systems [1], [2]. Among these frameworks, Boolean Genetic Regulatory Networks (GRN) introduced more than forty years ago provide a convenient qualitative formalism [3], [4], which has since been the subject of numerous theoretical studies and extensions [5], [6]. Boolean GRNs, including their generalisation to account for multi-valued variables [7], have proved useful for modelling and analysing regulatory and signalling networks for which precise quantitative data are often scarce (see *e.g.* [8]–[13] for this framework applied to cell cycle modelling).

Briefly, a Boolean GRN is defined by a signed, directed graph, where the nodes represent genes (or more generally regulatory components) and signed edges represent the regulatory interactions between these components: positive (resp. negative) edges denote activations (resp. inhibitions). Each node is associated with a Boolean variable that accounts for the expression state (ON/OFF) of the corresponding gene, and a logical function specifies the evolution of this variable, depending on the variables associated with the regulators of the gene. More precisely, at each time step, gene values are updated according to the results returned by their logical functions. There is a variety of Boolean GRN models that differ in their classes of logical functions (*e.g.* additive, canalizing, unrestricted), in their structural properties (*e.g.* fixed, bounded or unrestricted indegrees), or in their updating scheme (*e.g.* synchronous, asynchronous, block-sequential).

To define a model, in addition to the already challenging problem of identifying the wiring of the (signed) regulatory network, one has to specify the logical functions associated to the nodes. That is to say to specify how regulatory effects are combined. In this context, some authors choose to rely on functions uniquely defined from the regulatory structure [8], [10], [14]. In particular, in Boolean threshold networks, regulatory effects are assumed to be additive: each function is defined as a majority rule where the decision to activate a gene follows from the comparison of the sum of the (possibly weighted) contributions from the regulators to a specific threshold. Boolean threshold networks have been successfully used to model the control of cell cycle [8], [10]. Zañudo *et al.* have performed a thorough study of random Boolean threshold networks defined as a subset of the ensemble of Kauffman's random Boolean networks, where regulators and regulatory functions are randomly chosen [15]. Finally, it is worth noting that Boolean threshold networks originate from the McCulloch-Pitts neural model [16], which gave rise to countless studies and applications.

To account for the inherent stochasticity of regulation processes, stochastic versions of Boolean GRNs have been proposed in the literature [17]–[22]. Schlumevitch and colleagues define Probabilistic Boolean Networks, where a set of regulatory functions is assigned to each gene and, at each time step, one function is randomly chosen within this set [17]. This setting results in dynamics that can be represented as a Markov chain. Other authors propose to update each gene according to its regulatory function with a given probability [18]–[21]. Garg *et al.* discuss this model they call Stochasticity In Nodes (SIN), indicating that it can lead to noise overrepresentation. They propose an alternate model, called Stochasticity In Functions (SIF), that differently accounts for the stochasticity of the function failure: it associates different failure probability to different logical gates and stochasticity also depends on the state of the regulators [22]. We finally refer to [23] for a seminal discussion of the complete probabilistic version of such models in the context of neural networks.

Here, focussing on threshold Boolean networks, we propose that the majority rule is particularly suitable to combine deterministic and probabilistic updates. Indeed, the combined contribution of the regulators at a given time is not always conclusive to enable an unambiguous choice of the gene evolution. Hence, we propose a stochastic tie-breaking that associates a probability to the update value when positive effects countervail negative effects. Furthermore, various majority rule settings can be devised that are specified and discussed in this paper. We extensively study a class of two gene networks, considering different majority rule settings. We show that this simple motif gives rise to a wide variety of behaviours and that the regulatory structure plays a role in the degree of stochasticity exhibited by the dynamics. We further revisit the Li *et al.*'s deterministic Boolean threshold model of the budding yeast cell cycle [8]. Interestingly, several studies have considered stochastic versions of this model, with intent to explore the model robustness (*e.g.* [18], [24], [25]). Here, we illustrate the interest of our approach to tackle this question. In particular, we demonstrate that steady state analysis can be rigorously performed and lead to effective predictions; these relate to the identification of interactions whose addition would ensure that a specific state is an absorbing state.

## Methods

Boolean Gene Regulatory Networks (GRN) are defined by a directed graph where the nodes represent the regulatory components (genes or their products) and the edges represent the regulatory interactions. We denote the nodes 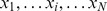 (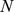, the number of nodes). Each node is associated with a level of expression (or of activity) referred to as 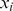 for simplicity. This level may change in time, taking the value 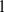 (ON) or 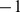 (OFF). An edge from 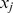 to 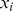 is denoted 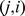 and is associated with a sign 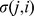, which is positive for an activation 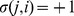 or negative for a repression 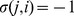. The source of the edge 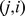 is thus a regulator of gene 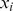. If 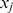 does not regulate 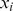 then 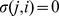.

The dynamics takes place in the configuration space 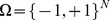 (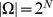) and configuration 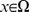 is defined by the values of the 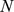 nodes: 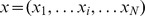.

The evolution of each node is defined by an *updating rule*, which depends on the regulators of that node and the time variable is discrete: 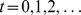. Note that there is an edge from 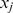 to 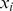 if, for some fixed values of the other regulators of 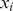, changing the value of 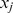 has an effect on the value of 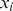 at the next time step: such regulatory interactions are said *functional* (*e.g.* [26]).

We first introduce the *Majority Rule* (MR) that, given the configuration of the system at time 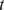, 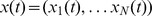, defines the configuration at the next time 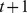:
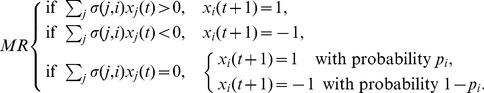
(1)


Hence, in Equation 1, an activator (resp. a repressor) has a positive contribution if it is present (resp. absent). When the sum of the contributions is zero (*i.e.* there are as many positive and negative contributions), rather to arbitrarily opt for a value, the MR sets 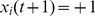 with probability 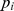 and 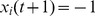 with probability 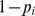. A node is *deterministic* if its updating rule is deterministic for *any* configuration, and *probabilistic* if its updating rule is probabilistic for *some* configurations. Therefore, in the case of the MR, a node is deterministic if it has an odd in-degree (*i.e.* an odd number of regulators) and probabilistic if it has an even in-degree.

If there is at least one probabilistic node, the dynamics of the model can be represented by a finite Markov chain on the configuration space 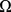; otherwise, we have a deterministic dynamical system in 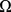. Extending the usual notion of absorbing chains [27], we say that the chain is absorbing if all ergodic sets are deterministic: either fixed points (*i.e.* configurations such that 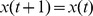 with probability one) or cycles (*i.e.* sets of configurations such that there exists a 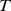 for which 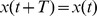 with probability one). Hence, with this definition, the set of absorbing states includes states that are members of deterministic cycles. It corresponds to the usual definition applied to a power of the transition matrix. Moreover, we will often refer to the terminology of the dynamical systems community by calling *attractors* the (minimal) ergodic sets of a chain, that are also defined as the terminal strongly connected components of the transition diagram.

For completeness, we also investigate two variants of the MR. The first variant, referred to as *Inertial Majority Rule* (IMR), considers the current state of a probabilistic node to define its next value in the case of equal number of positive and negative contributions:
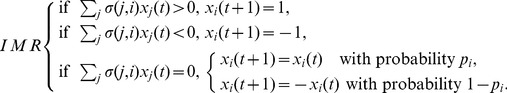
(2)


We designate this rule *inertial* because its deterministic version (when 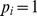) specifies that nodes keep their current values when activations and repressions cancel each other out. It is worth noting that this rule amounts to adding a functional self-activation on each node: when the sum of the contributions from all other regulators is zero, it is the value of the proper node that determines its next level.

In the next MR variant, referred to as *Null Majority Rule* (NMR), the nodes take values 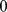 and 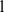. Hence the configuration space is 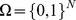 and we denote 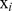 the level of the 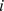
th node, to distinguish from 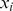, which takes values 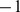 and 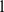:
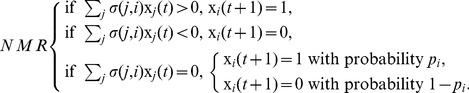
(3)


Hence, under the NMR, when the level of a regulator is zero, it plays no role in the regulatory function. As a consequence, whatever the sign of the interaction (activation or inhibition), the absence of a regulator results to the same (lack of) contribution in contrast to the MR, where e.g. the absence of a repressor has a positive contribution. Importantly, whatever their in-degree, all nodes are probabilistic.

These two variants of the majority rule can be combined in an Inertial Null Majority Rule (INMR) as in the model of the cell cycle control in yeast specified by Li *et al.* [8] (see below, the section devoted to the yeast cell cycle model).

Because the evolution of any node only depends on its regulators, it will be convenient to focus on structures that we call *modules*, which are composed by one node 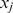 and its incoming interactions.

Finally, it is worth noting that the majority rules defined above are special cases of the regulatory functions considered in threshold Boolean networks, where the sums of contributions include interaction weights 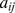 (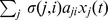) and compare to activation thresholds 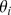 [15]. Here, all interaction weights are set to 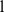, and all thresholds are zero.

## Results

### Two-node Gene Regulatory Networks

Here, we consider connected Gene Regulatory Networks (GRNs) encompassing two nodes 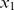 and 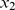. There are three classes of such two-node GRNs that include respectively two, three and four interactions. The first class contains three elementary cross-regulatory circuits; two circuits are positive circuits (*i.e.* the product of the interaction signs is positive) and one circuit is negative with a node activating its repressor. There are indeed two such circuits which are equivalent up to exchanging node labels: 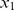 activates 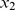, which inhibits 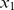 or 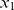 inhibits 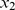, which activates 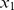. In these models, both nodes are deterministic under the *Majority Rule* (MR). The second class encompasses the networks made by cross-interactions and a single self-interaction (six such networks, up to exchanging node labels). Under the MR, the self-regulated node is probabilistic, whereas the other node is deterministic. These models give rise to: 1) bi-stable dynamics (when both circuits are positive), 2) an absorbing period-2 cycle (when the cross-regulatory circuit is positive and the self-regulation is negative) and 3) combination of cycles over the four configurations (when the cross-regulatory circuit is negative).

We choose to thoroughly study the third class, for which both nodes are probabilistic. We thus consider all the GRNs defined by cross-interactions between nodes 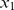 and 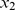, which are both self-regulated (for convenience, we use free variables 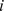 and 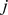 such that 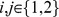 and 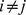). We start by considering the MR. Then, we point out the differences with the inertial and null MR variants (IMR and NMR).

We denote by 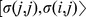 the module where 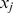 is self-regulated (with sign 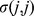) and is regulated by the node 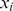 (with sign 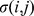); there are four modules of this type. We are thus interested in the networks that result from the composition (denoted 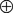) of two such modules.

In what follows, the Markov transition matrices 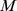 are 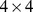 matrices with entries corresponding to configurations 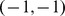, 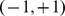, 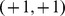, 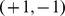 (in this order, which facilitates the description of the rotation that transforms one model into another, see below).

**Figure 1:**
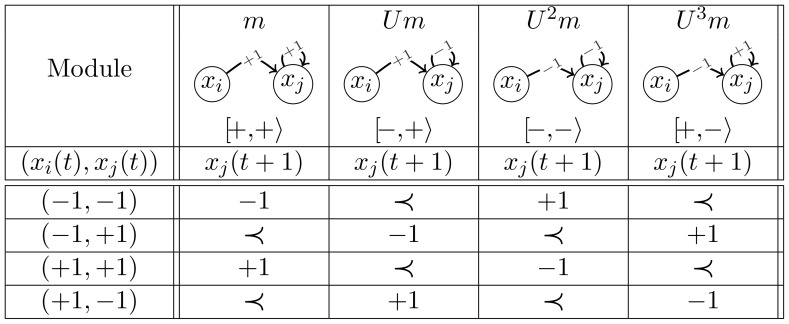
The four modules and their evolutions for the majority rule (MR). The sign 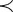 corresponds to the probabilistic choice: 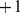 with probability 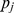 and 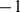 with probability 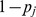. In each column, 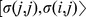 is the symbol associated to the module (sign of the self-regulation and sign of the cross-interaction).

Figure 1 summarises the dynamical rules for the four modules, considering the MR as defined by Equation 1. There are 16 models corresponding to the different combinations of two modules. Notice that a row rotation (modulo 4, from top to bottom) transforms each module (column) into the next one. Denoting by 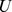 this transformation and arbitrarily denoting by 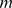 the 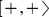 module, we refer to the remaining modules as indicated in Figure 1: 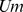, 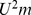 and 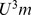 (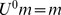).

We first observe a node symmetry that relates 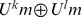 and 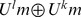 by exchanging 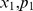 and 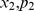. Referring to the relation between the two modules that define a two-node GRN, we partition the set of the models 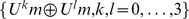 in two subsets: eight models are said *in phase* (IP), when 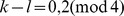, that is when the probabilistic choices are located in the same row in Figure 1; the remaining eight models are *out of phase* (OP), when 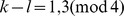. In the former case (IP), the Markov matrix has two rows with four probabilistic entries each combining the two parameters (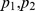) and two rows with a deterministic entry (*i.e.* with probability one). This defines 10 transitions in the corresponding dynamical diagrams. Whereas in the later case (OP), each row has two probabilistic entries (either 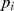 or 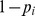), giving rise to eight transitions in the dynamical diagrams.

We search for other symmetries to reduce the case studies of our two-node models. From a mathematical standpoint, which does not always fit the functional perspective, two models are equivalent when their Markov matrices are the same up to a renaming of the state space and a bijective correspondence of the parameters 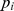. Clearly, a necessary condition for this equivalence is that the diagonal elements of the matrices are the same up to parameter exchanges. In particular, an IP model cannot be isomorphic to an OP model. By inspection of the diagonal entries of each model and elementary computations, we end up with a complete classification of all the models.

There are eight IP models grouped into three isomorphic classes, IP1, IP2 and IP3. They are characterised by the existence of two deterministic transitions whose specific locations govern the dynamics of the model. There are also eight OP models grouped into three isomorphic classes, OP1, OP2 and OP3. Contrary to the IP models, all the transitions are probabilistic and depend on only one of the parameters (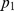 or 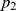), allowing a complete flexibility of the mean visit times associated to each connected component of the dynamical graph.

#### Model class IP1

It includes the two models 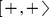 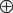 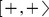 (*i.e.* 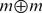) and 0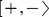 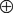 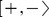 (*i.e.* 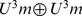). From the structural symmetry point of view, this class contains the models with self-activations and symmetrical cross-interactions (*i.e.* positive two-node circuits). The transition matrix of 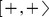 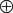 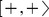 is:
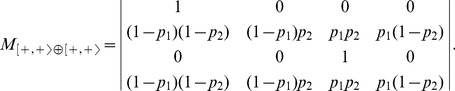
(4)


**Figure 2:**
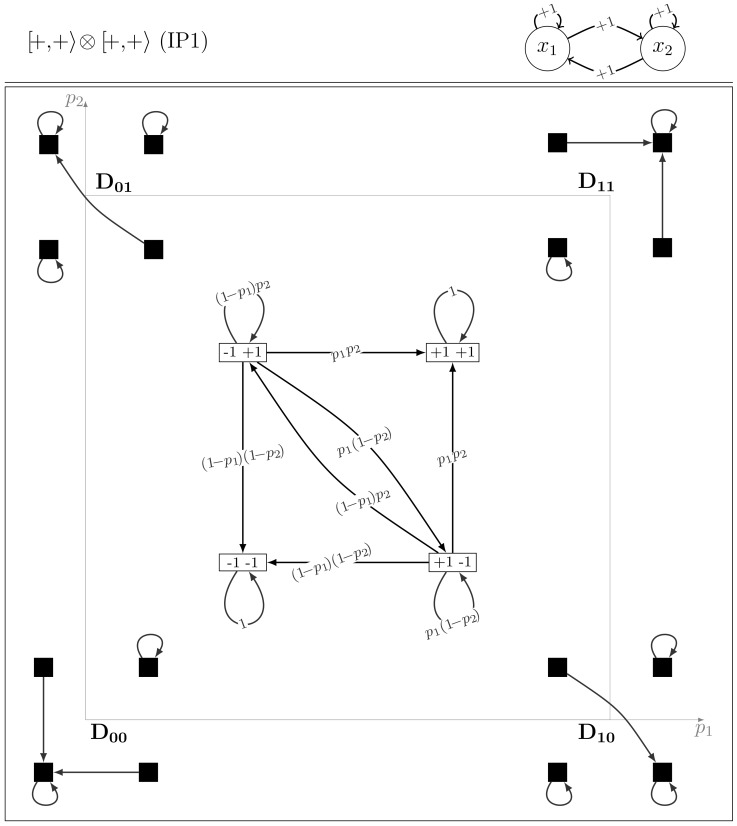
The dynamics of the IP1 model 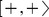 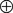 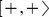.

The model 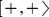 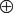 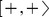 together with its dynamics depending on the values of the parameters 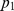 and 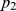 are depicted in Figure 2. The transition matrix of 0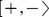 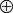 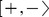 can be deduced from the matrix of 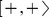 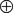 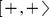 by permuting the entries 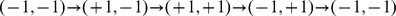 and changing 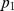 to 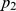 and 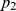 to 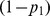:
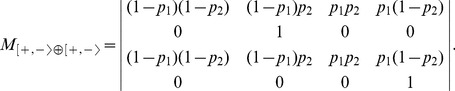
(5)


Therefore the dynamics of 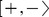 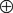 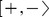 is isomorphic to that of 0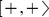 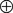 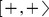. These models are self-symmetric by node symmetry. The two deterministic transitions (*i.e.* with probability one) are loops on single states (*i.e.* diagonal elements in the transition matrix). In other words, the corresponding Markov chains are absorbing with two fixed point attractors. The fundamental matrix is [27]:
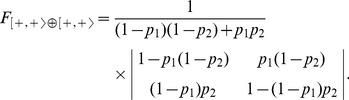
(6)where the first entry is for 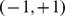 and the second for 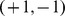.

Recall that the fundamental matrix 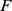 of an absorbing chain is defined as the inverse of the matrix 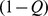, where 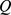 is the sub-matrix of the transition matrix 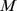 restricted to the set of transient states [27]. Entry 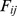 of the fundamental matrix has a nice probabilistic interpretation: it corresponds to the mean time spent by the process in configuration 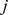 if it starts in 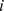. Note that this value is finite because 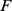 is defined on the transient states. Relying on our extended notion of absorbing chains, when ergodic sets are deterministic cycles, we can similarly define a fundamental matrix and use the same rationale by simply considering a power of 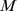 instead of 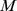.

Therefore, starting in the configuration 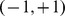 (or 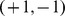), for typical values of the parameters around 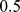 (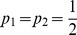), the mean time spent by the process in one of the transient configurations is of order one (actually 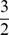). It diverges when the parameters tend to opposite extreme values (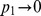, 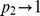) or (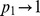, 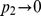), where at the limit, a third fixed point appears. Instead, when both parameters are close to 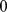 or 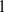, the dynamics still encompasses two absorbing configurations, while expected times to reach these configurations tend to 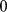 or 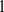.

When 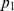 and 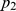 are fixed to their extreme values (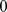 or 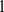), the system is deterministic, and the rules governing the evolution of the nodes can be defined by means of logical connectors. Here,

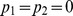
 corresponds to an AND rule on both nodes (the presence of the two activators is required to reach level 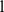);

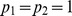
 corresponds to an OR rule on both nodes (the presence of at least one activator is required to reach level 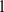);

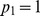
, 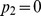 corresponds to an OR rule on node 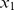 and an AND rule on node 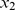;

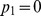
, 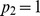 corresponds to an AND rule on node 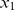 and an OR rule on node 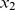.

A remarkable feature of this type of models is its ability to continuously exchanging two logical connectors by weighting the respective probabilities of implementation. For instance when 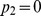, 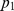 is the probability to activate the dynamical connection corresponding to an OR rule on node 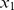 and 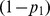 is the probability corresponding to an AND rule. This is clearly illustrated in the dynamical graphs in Figure 2. In this sense, we can say that the border of the parameter domain constitutes a continuous family of *Stochasticity In Functions* models (SIF) following the definition in [22]. The whole parameter domain can thus be seen as a generalisation of these stochastic models, also corresponding to the probabilistic Boolean networks proposed by Schmulevich *et al.* [17].

In fact, by a theorem on random map realisations of Markov chains (see [28], chapter 1.2), our two-node models can be realised as random walks on the set of the dynamical graphs of the four extreme models (*i.e.* for which the parameters 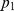 and 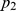 equal 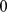 or 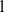). Let us denote these dynamical graphs by 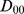 (for 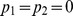), 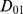, 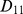 and 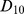 (see Figure 2). Notice that, in the dynamics of these deterministic models, any configuration has a unique outgoing transition. At each time step, one extreme model is randomly and independently selected and the next configuration is chosen according to the (unique) transition leaving the current configuration of the corresponding dynamical graph. 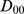 is taken with probability 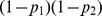, 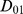 with probability 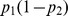, 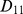 is taken with probability 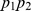 and 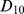 with probability 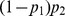. This random walk has exactly the same probabilistic transitions as the original IP1 model depicted in Figure 2.

#### Model class IP2

It includes the two models 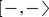 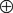 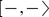 and 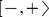 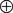 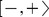. From the structural symmetry point of view, this class contains the models with self-inhibitions and symmetrical cross-interactions (*i.e.* positive two-node circuits).

**Figure 3:**
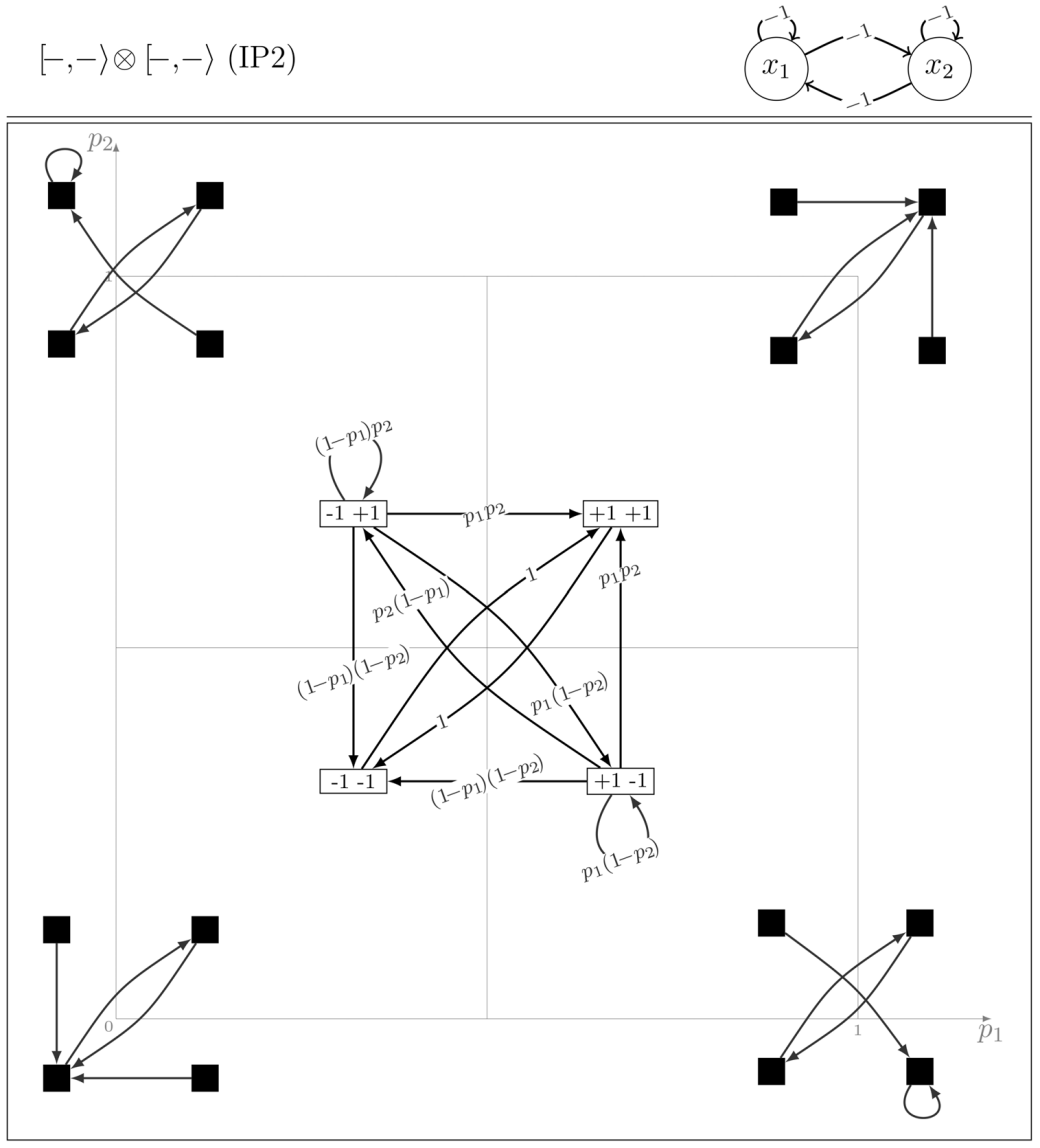
The dynamics of the IP2 model 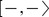 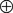 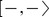.

The model 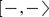 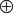 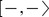 is changed into 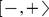 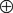 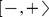 by permuting the entries 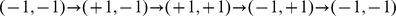 and changing 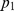 to 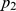 and 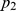 to 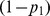. The two models are also self-symmetric by node symmetry. Because the two deterministic arrows (*i.e.* with probability 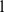) interchange two states, the corresponding Markov chains are absorbing with a unique attractor, a period-2 cycle (see Figure 3). Therefore, regardless the initial configuration, all the realisations end up in this cycle, with probability one.

Because 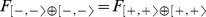, the transient dynamics of 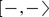 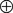 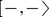 and of 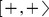 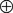 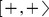 are identical and the analysis of the parameter space follows along the same lines as for the previous class.

#### Model class IP3

It includes four models: 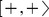 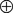 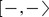, 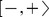 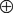 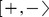, and their homologous node symmetric 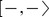 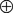 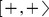 and 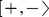 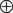 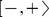. From the structural symmetry point of view, this class contains all the models asymmetrical with respect either to the self-interaction or to the cross-interactions. By permuting the entries: 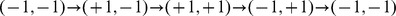 and by changing 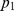 to 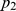, 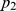 to 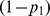, 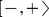 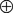 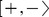 is changed in 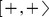 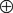 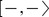. Notice that an IP2 model cannot be isomorphic to an IP3 model, even if they share the same diagonal elements. This is because, in the IP2 class, the deterministic arrows deal with two states while in the IP3 class, four states are concerned and this property is invariant by isomorphism of the state space. IP3 models define regular chains (the four states constitute a unique ergodic set, unless the parameters take extreme values), but the presence of the two deterministic transitions put an extra weight on the correspondent target states.

**Figure 4:**
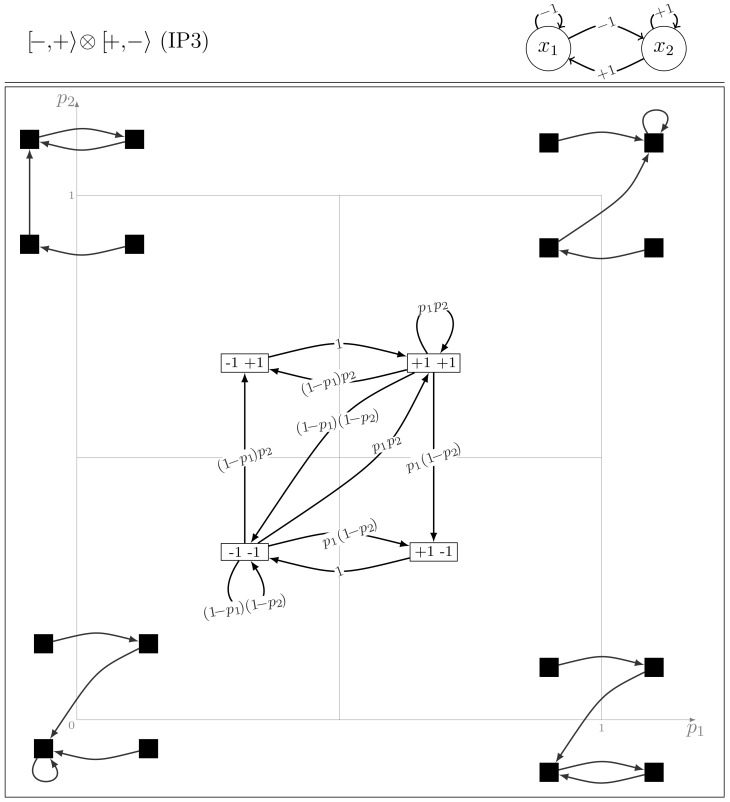
The dynamics of the IP3 model 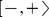 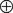 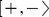.

Figure 4 shows that there are many cycles, giving rise to oscillations that can visit any configuration in any order and with different return times. The mean return times to each configuration 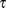, kind of a mean period of the oscillations, can be computed from the invariant probability distribution and reads: 
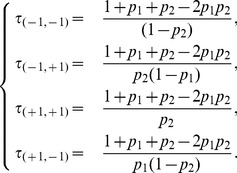
(7)We recall that 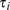, the mean time taken by a regular chain that starts at 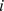 to return to its starting point (the mean return time at 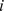), is given by the inverse of the 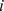
th component of the limiting probability vector (see [27], Theorem 4.4.5). It is also possible to compute this value using the fundamental matrix of the process ([27], Theorem 4.4.7). Note that, for a regular Markov chain, the definition of the fundamental matrix slightly differs from that of an absorbing chain (see [27], Definition 4.3.2).

**Figure 5:**
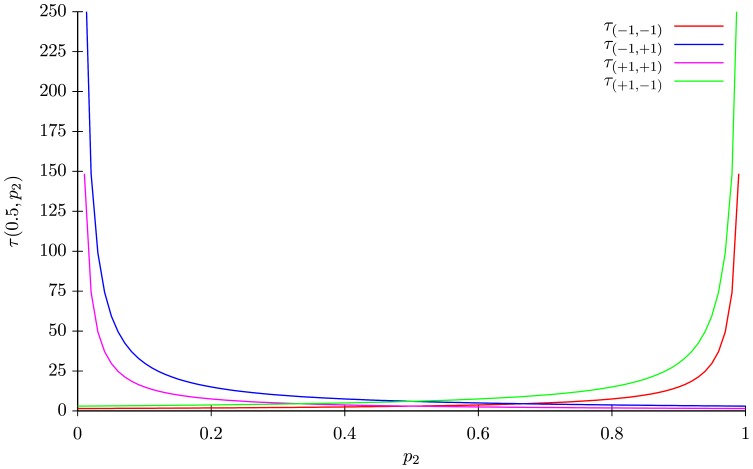
Mean return times of the IP3 model 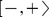 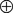 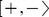 as a function of 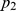, with 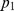 fixed to 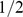. In this plot, one can observe for instance, that the mean return times to 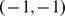 varies from 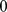 (when 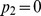) to 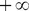 (when 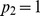).

In Figure 5, the values of the mean return times 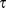 are depicted as functions of 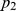, for 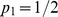. Not surprisingly, due to the deterministic transitions, the mean return time to 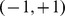 (resp. 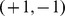) is always larger than that to 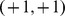 (resp. 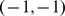). When 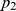 tends to an extreme value, the system turns into an absorbing chain and the return times of the transient configurations diverge. As for the other IP models, the extreme cases correspond to models where rules are defined by means of logical connectors. Hence, Figure 5 is a further illustration of a continuous parameter swap between different logical rules.

#### Model class OP1

It includes 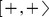 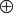 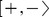 and its node symmetric counterpart 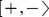 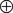 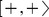. From the structural symmetry point of view, the class contains all the models with self-activations and asymmetrical cross-interactions. OP1 models are the probabilistic counterpart of the negative circuits studied in [29]: the dynamics is built on a fundamental period-4 cycle combined with fluctuating sojourns in each configuration. The transition matrix 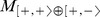 is:
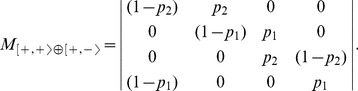
(8)


**Figure 6:**
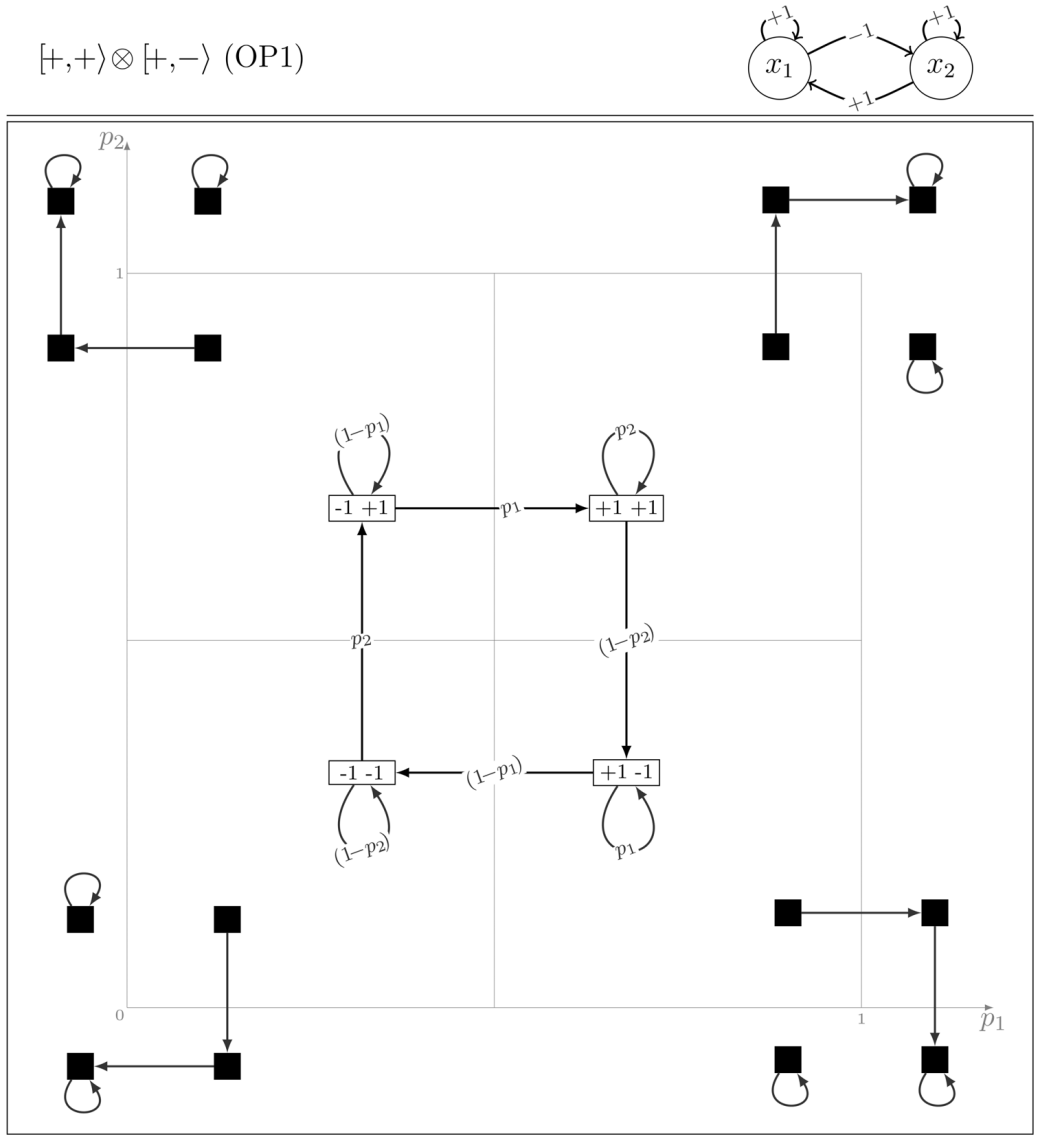
The dynamics of the OP1 model 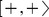 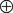 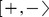.

Figure 6 illustrates the relevant features of the dynamics of this model. Notice that, by changing the parameters, it is possible to modulate the time spent in each configuration and therefore the mean period of the oscillations. This observation is corroborated by the computation of the mean return times:
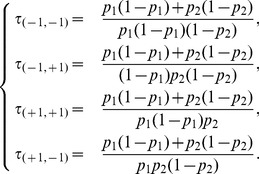
(9)


For extreme values of the parameters, the system is bistable.

#### Model class OP2

It includes the two models 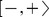 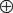 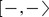 and its node symmetric counterpart 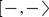 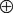 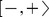. From the structural symmetry point of view, the class contains all the models with self-inhibitions and asymmetrical cross-interactions (negative circuits between the two nodes).

**Figure 7:**
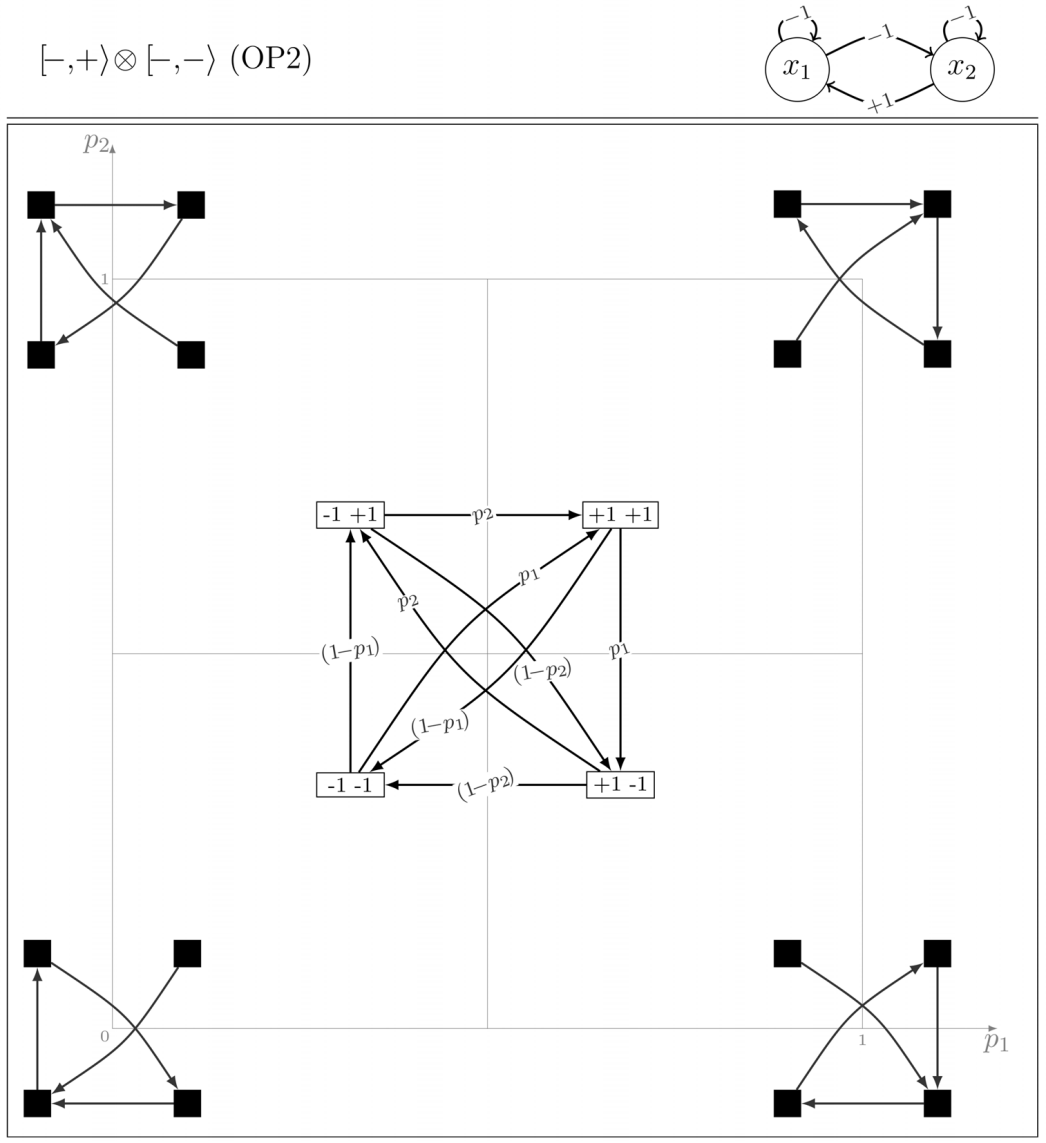
The dynamics of the OP2 model 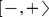 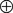 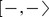.

Figure 7 shows the existence of synchronous transitions where both nodes change simultaneously their values, inducing various period-2, 3 and 4 cycles. Combinations of these cycles lead to oscillations of any order. The extreme cases display four deterministic periodic dynamics, each including one synchronous transition that involves simultaneous updates of the two nodes. The analytical expressions of the mean return times are:
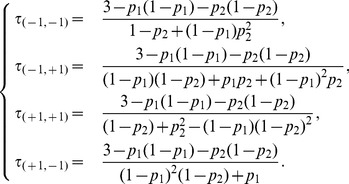
(10)


The set of equations (10) fully supports the idea of continuous parametric transitions among these dynamics: while the probability of a period-3 cycle increases as parameters tend to their extreme values (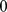 or 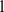), for intermediate parameter values, higher values of 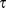 indicate that the period-4 orbits become prominent.

#### Model class OP3

It includes four models, 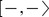 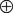 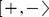 and 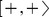 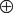 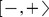 and their node symmetric counterparts. From the structural symmetry point of view, the class contains all the models with self asymmetrical interactions and symmetrical cross-interactions. By permuting the entries: 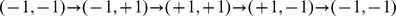 and changing 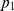 to 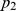 and 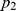 to 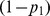, model 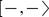 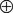 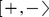 is changed into 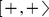 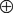 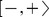. The dynamics of these models alternate chains of period-1 to 4 cycles. It may thus be viewed as a transition between OP1 and OP2 models.

**Figure 8:**
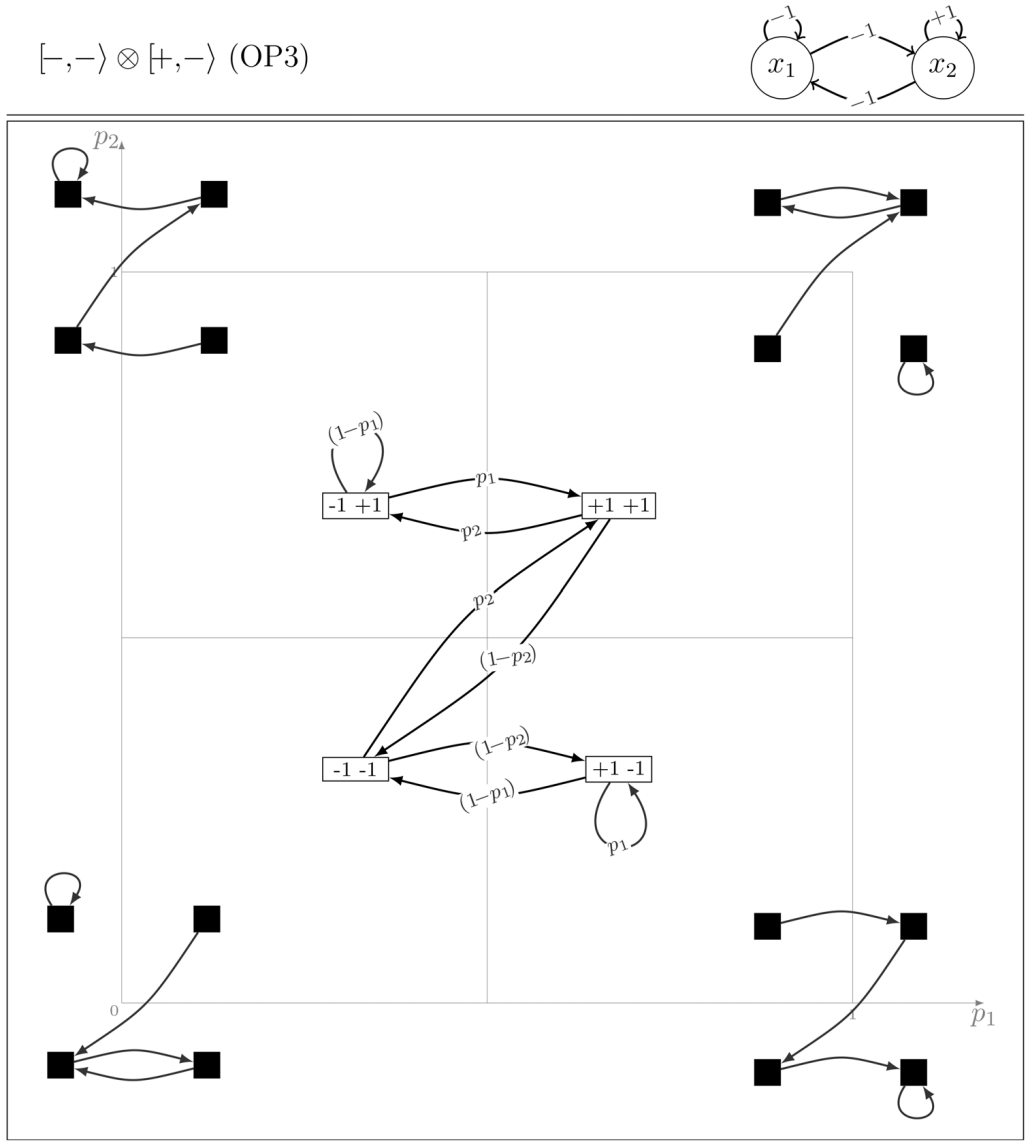
The dynamics of the OP3 model 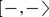 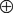 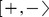.

Figure 8 exhibits the dynamical properties of this model. In particular, in the extreme cases, we observe the existence of deterministic fixed points possibly combined with a period-2 cycle.

The existence of oscillations of any period is also shown in Figure 8 and Equation (11) points to a large variety of time scales of the oscillations when parameters are changed:
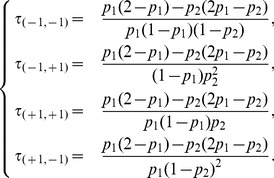
(11)


### Two Majority Rule variants

Here, we briefly analyse the cases of the two variants previously introduced: the Inertial Majority Rule (IMR) and the Null Majority Rule (NRM).

#### The Inertial Majority Rule

**Figure 9:**
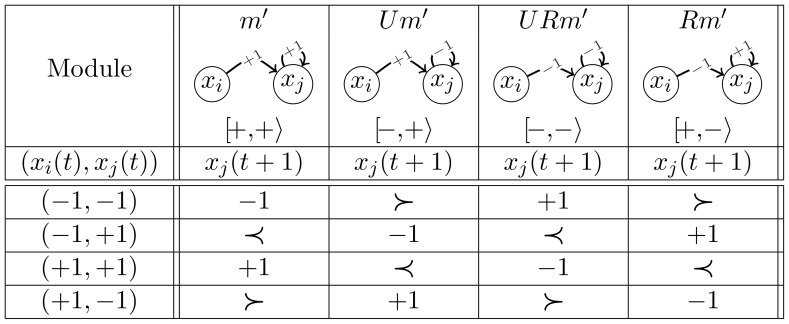
The four modules and their evolutions for the Inertial Majority Rule (IMR). The sign 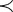 corresponds to the probabilistic choice: 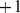 with probability 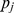 and 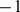 with probability 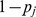 whereas the sign 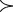 corresponds to the opposite probabilistic choice: 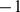 with probability 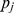 and 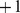 with probability 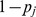. 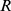 is a reflection, 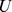 is a rotation as for MR in Figure 1.

This rule defines that, whenever activations and repressions cancel each other out, the next level of a node depends on its current level (Equation (2)). For our two-node models under the IMR, we can define the same isomorphism classes as those of the MR. From Figure 9, one can observe that the symmetry for the IMR is slightly different from that of the MR. There are two types of probabilistic choices, introducing a row reflection 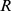 besides the rotation 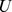 to relate the modules. For example, 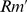 evolution in Figure 9 is obtained by rotating module 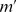 rows (transforming 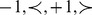 into 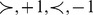). As a consequence, the isomorphism between models under the IMR relies on a different parameter change when compared to the MR: 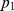 is changed to 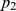 and 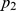 to 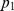. However, IMR and MR have exactly the same model classes and similar dynamics. Only differences regarding transition probabilities arise for the models combining an even and an odd module, *i.e.* an even and an odd column of Figure 1 (for the MR model) and Figure 9 (for the corresponding IMR model). For instance, in the case of the OP3 model 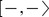 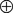 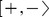, defined by the third and fourth columns of Figures 1 and 9, the two loop transition probabilities are different for the MR (namely 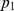 and 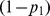), whereas they are identical for the IMR (namely 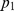). The probabilities of the transitions connecting configurations 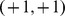 and 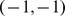 similarly differ between the MR and the IMR. The reason for this clearly appears in the Figures 1 and 9 where the probabilistic choices are identical in both columns for the MR whereas they are opposite for the IMR.

#### The Null Majority Rule

The majority determined under the NMR is quite different as compared to that of the MR and the IMR (see Equation (3)). Indeed, a node whose level is 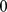 has no contribution in the updating decision of its targets. Still, one can define a bijection between both representations. In any configuration, let 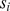 denote the (global) contribution of the regulators targeting node 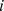 (*i.e.* 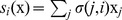). We have:
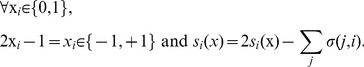
(12)


Note that the very same change of variables was defined by F. Robert, coming up with two equivalent formulations for threshold networks [30]. However, to ensure equal dynamics, the threshold functions and the thresholds were accordingly modified. Here, our purpose is different and amounts to revising the semantics of repression contributions (therefore the zero threshold is maintained for all the nodes).

**Figure 10:**
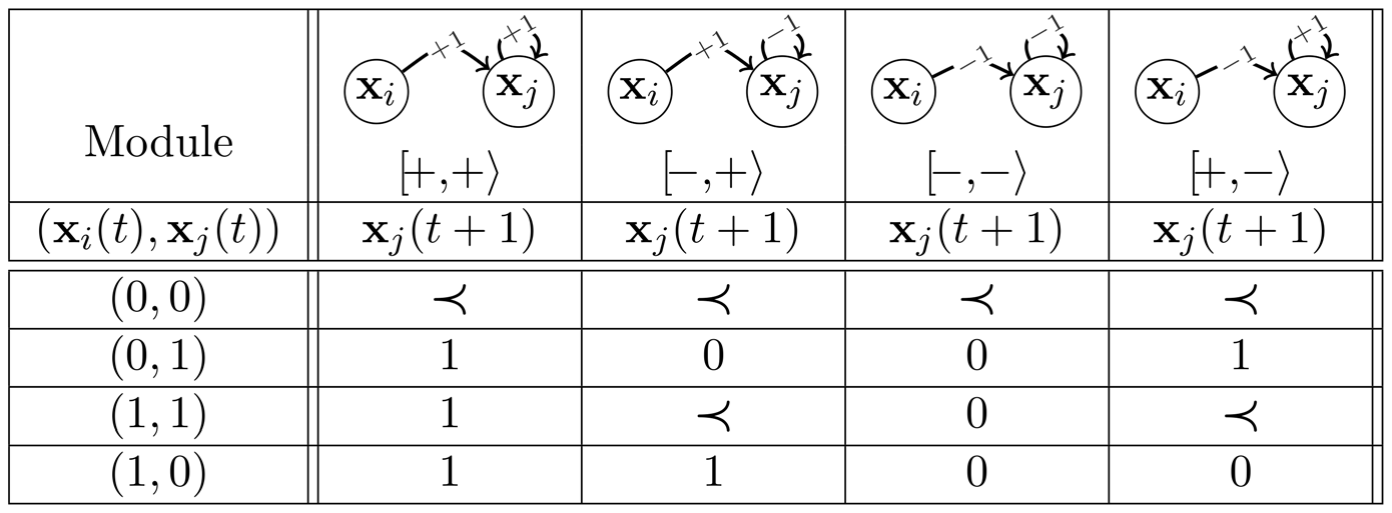
The four modules and their evolutions for the null majority rule (NMR). The sign 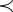 corresponds to a probabilistic choice: 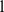 with probability 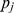 and 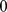 with probability 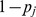. Node levels take values 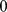 and 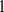.

The modules 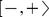 and 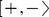 are identical under the MR and NMR because, in these cases, 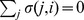 (see Figure 10). As a consequence, the four NMR models built with these modules have the very same dynamics as their MR counterparts. Moreover, considering the NMR, if at a given time, 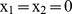, then 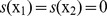 and the sixteen models have the same probabilistic updating for this configuration. Finally, it is easy to check that starting at time 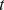 from the remaining configurations 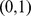, 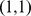 or 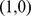, the updating process leads to 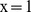 in the module 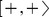 and 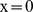 in 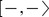. From these observations, it turns out that NMR models have more deterministic transitions than their MR analogs. Not surprisingly, there are thus more absorbing models under the NMR than under the MR. This is a remarkable difference from the biological perspective since under the NMR, in eleven out of sixteen models, the dynamics converge to a fixed point or a small cycle. Hence the NMR displays robust, restricted behaviours. Moreover, changes in parameters values only impact times for convergence to attractors whose identities are conserved. In contrast, the MR is more flexible, leading to models with a larger variety of behaviours.

Finally, with the INMR that results from the combination of the inertial and null majority rules, the module evolutions are similar to those defined in Figure 10, except that for configuration 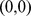, 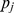 and 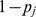 are interchanged (*i.e.* for all the modules, value 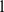 is chosen with probability 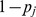 and 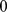 with probability 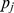).

### The yeast cell cycle network revisited

#### The original model

**Figure 11:**
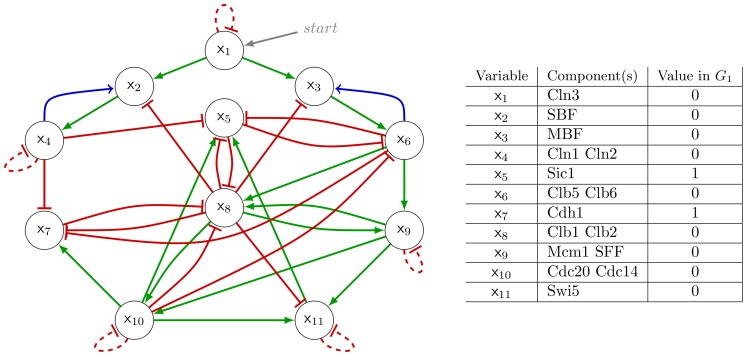
The yeast cell cycle model as defined in [8]. Green arrows denote activations, whereas red T-ended edges denote inhibitions. In Li *et al.*'s model, self-degradations (dashed red loops) were added to the nodes that have no negative regulators. When considering the stochastic MR rule, these self-loops can be discarded (see text). With the addition of the two activatory edges in blue, 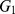 becomes the unique attractor of the model when 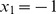. The table on the right indicates molecular counterparts of nodes 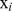 as well as their values in the 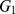 configuration.

The eukaryotic cell cycle defines a series of phases undergone by cells that divide, giving rise to daughter cells. G1 is a growing phase, known as gap 1 phase, followed by the S phase of DNA synthesis and chromosome replication. Then, after the gap phase G2, the M phase proceeds with the separation of the chromosomes and culminates with cell division. In [8], Li *et al* define a Boolean Gene Regulatory Network that encompasses the main regulators of the cell cycle progression in the budding yeast. The network supporting this model is depicted in Figure 11. The authors use a deterministic Inertial Null Majority Rule, hence the 11 variables 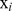 take values 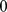 or 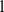, and 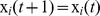 when 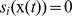 with probability 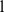. Interestingly, Davidich and Bornhold's Boolean model of the fission yeast cell cycle uses the very same rule [10]. Recently, Fauré and Thieffry describe and compare logical models of the molecular networks controlling the cell cycle in different eukaryotic organisms [11].

Cyclin Cln3 is known to be crucial for the cell commitment to S phase, *i.e.* for the cell cycle progression. In this model, Cln3 (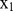) thus acts as an input of the network (possibly stimulated by a *start* signal). As a key feature, the model has a fixed point denoted 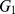, which corresponds to the G1 phase and that attracts most of the trajectories, considering all possible initial conditions. There are other six fixed points in the model, but those have a rather restricted basin of attraction and no meaningful biological counterparts. Moreover, starting from the state 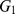, and artificially switching Cln3 ON, the model follows a trajectory matching the cell cycle progression until reaching back the state 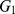.

Li *et al* considered the large size of the basin of attraction of the biological fixed point 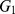 as a good indication of the robustness of the network to perform its function. This is confirmed by showing that the size of this basin of attraction is mostly preserved under perturbations that randomly remove or introduce a regulatory interaction. In [25], Stoll *et al.* propose another type of perturbations: 1) shuffling the wiring yet keeping the connectivity at each node or 2) removing one to several regulatory interactions. Using Li *et al.*'s model as a case study, they consider the size distribution of the basins of attraction and distance to a reference attractor as useful measures to assess impact of these perturbations. Zhang and colleagues assess the effect of stochasticity on the Li *et al.* model by turning it to a probabilistic model where all transitions in the configuration space are made possible [18].

In the framework of the present work, it is natural to consider the model described above as an extreme case of its stochastic version and to study the robustness of the dynamical behaviour faced to perturbations in the probability parameter space.

Therefore, we consider the stochastic version of this model under the Inertial Null Majority Rule: when 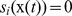 (the sum of the contributions is zero) we have 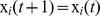 with probability 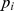, otherwise 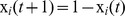 with probability 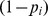. As for the two-node models under the NMR, all the modules are probabilistic. In particular, when all the node values equal zero (see Figure 10). In the configuration 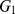, all genes are inactive but 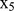 (which negatively regulates 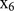 and 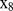) and 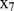 (which negatively regulates 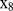). We have thus that 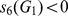, hence 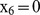 is stable in 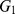, similarly for 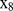. Consequently, 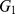 is not absorbing, except if 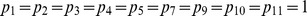. When these parameters are closed to 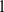, the system may be steady in 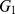 long enough to match the biological situation, but it will eventually (after a finite time, with probability 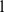) leave 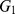, following a trajectory different from the cycle described in [8].

In the deterministic case, the INMR favours the existence of steady states including those with active genes whose regulators are all inactive; as discussed in [15], the fact that a node keeps its current value when the sum of the contributions is zero leads to frozen nodes. As already mentioned, the inertial rule amounts to add a self-activation on every node. It is worth mentioning that the self-inhibitions of the model (see Figure 11) are not *functional* (see [26]), they merely cancel out these self-activations, which are hidden in Li *et al.*'s model. In other words, for nodes that are only positively regulated, the NMR is applied.

In contrast to the deterministic INMR, the stochastic model does not display such a stability. The aforementioned property of the inertial deterministic rule that generates frozen nodes does not hold anymore. In particular, when regulators are absent, activations and inhibitions are not discriminated, giving rise to a large number of probabilistic configurations. This is the main reason why 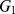, together with the other steady configurations of the INMR model, are not robust to the stochastic extension and are not absorbing states.

#### The model revised, considering the stochastic MR

We now consider Li *et al.*'s model under the stochastic MR as defined by Equation (1). Node values are thus set to 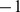 or 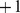 (and denoted by 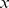 rather than 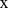). We recall that when the sum of its input contributions equals zero (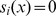), 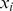 takes the value 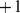 with probability 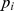 and 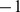 with probability 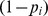.

In order to analyse the dynamical features of the model, in particular regarding its steady states, we take advantage of the combination of deterministic and probabilistic operation modes. As we shall see, the deterministic part of the dynamics imposes strict restrictions that are worth to inspect prior to follow up the study. We describe the strategy in some detail because it can be easily generalised and thus used to study any model under the same rule.

Recall that a configuration of the module 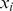 includes the values of all the regulators 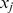 of 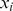. Beside the input node 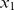, the yeast cell-cycle network has five deterministic modules, *i.e.* with odd in-degree, the remaining five being probabilistic. For a probabilistic module 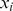, only configurations such that 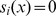 have a probabilistic outcome. An absorbing configuration 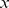, *i.e* for which 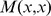, the element of the transition matrix equals 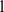, verifies:
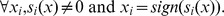



We first search for the steady configurations of the deterministic modules (they strongly restrict the number of candidates of absorbing configurations). Among the 32 configurations of the five deterministic nodes, we easily end up with only two candidates. All the other 30 configurations are discarded because they are not steady for at least one deterministic module. These two remaining configurations, steady for all the five deterministic modules, are 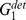 = (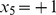, 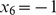, 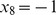, 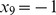, 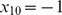) and 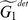 = (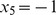, 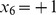, 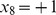, 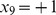, 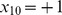). The former matches the biological fixed point 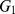 for the five deterministic modules, and the latter corresponds to its mirror image. Notice that the existence of these two solutions is a consequence of the correspondent symmetry of the MR (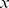 *versus* 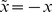).

We then look for all the possible extensions to the remaining six probabilistic nodes of these two solutions. The number of such extensions may be reduced if the values of the deterministic regulators of a probabilistic module determine the value of the corresponding node. Because 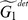 implies that 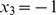, which is not compatible with 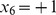, we conclude that 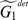 has no steady extensions.

Let us now explore the possible steady extensions of 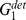. Recall that 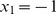 in 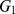. Clearly, from the already known inputs of module 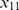 (that are 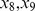 and 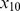), it follows that 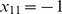. Looking now to the five known values for module 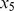 (*i.e* 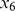, 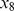, 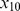, 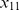 and 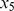 itself), we conclude that 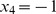, which in turn implies 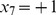. It remains to investigate 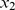 and 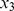. In order to have 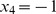 with non-zero probability (in fact 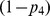) we should have 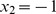. For module 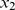, we have 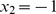 with probability 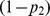. On the other hand, in order to be consistent with the values already fixed for module 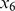, we need to set 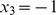, which is the case with probability 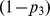. Therefore 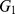 is steady with probability 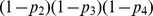. Remarkably, this analysis shows that the only steady configuration is 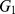, even if it is not absorbing; no other configuration remains steady with a non-zero probability.

This encouraging result naturally leads us to search for minimal changes in the interaction network that would turn 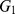 into an absorbing configuration. The first simple modification consists in eliminating the self-inhibition of 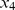, making this module deterministic with the proper outcome. Note that, because the MR accounts for the absence of a regulator, we could safely clean up the model by discarding the self-inhibitions of 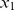, 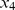, 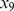, 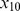 and 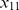. These were artificially added in the original model to ensure self-degradation of components that are not subject to other inhibition, under the INRM, and their elimination does not modify the results presented here. It remains the drawback of modules 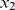 and 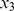. They can be fixed with probability one either by adding a positive interaction from a node whose values is 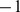 in the configuration 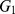, or by adding a negative interaction from a node whose value is 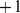 in 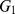. Interestingly, a modification that fulfils these constraints was mentioned by Fauré and Thieffry who propose to account for biological data suggesting that Cln1/2 and Clb5/6 positively their own transcription factors [11]. Adding these positive interactions from 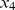 to 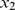 (Cln1/2 to SBF) and from 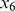 to 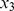 (Clb5/6 to MBF), 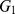 is the only steady configuration that turns out to be absorbing, that is to say to have a maximal robustness in the Markov chain context.

A subsequent question arises that concerns the existence of other absorbing trajectories in this modified model. By generating the state transition diagram of the corresponding Markov chain, we could verify that, when 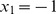, the 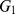 state is the unique attractor and thus, as mentioned above, for 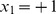, it is easy to deduce that the unique attractor is 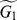, the mirror state of 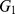. Hence, with probability 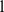, the system will reach either 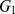 or 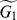, depending on the value of the input node 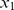. We have thus a full characterisation of the asymptotical behaviour of the model.

In this section, the cell cycle model of Li *et al.* has been used to illustrate the interest of our stochastic majority rule. Detailed biological interpretation of the model properties and further study to assess transient behaviours go beyond the scope of this paper.

## Discussion

In this work, we have presented a stochastic extension of threshold Boolean networks that includes both deterministic and probabilistic rules. In contrast to other studies where all transitions are made stochastic (*e.g.* [18]), a probabilistic choice is made only when the sum of the contributions equals the threshold (often set to 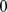), otherwise, the update is deterministic. This is rather natural from the biological view point. Indeed, it is reasonable to assign a probability to the update choice when regulatory effects cancel each others.

The originality of this model lies in the coexistence of deterministic and probabilistic nodes (or modules) in the same gene network; the former have a deterministic outcome for any input configuration, while the latter have probabilistic choice in certain configurations. This natural ambivalence open new interesting dynamical characteristics, yet avoiding a useless combinatorial explosion of trajectories. This point allows a rigorous analysis of certain dynamical properties of the model. In particular, we have shown how all the steady configurations may be identified and their properties modified in agreement with biological observations. More specific features of the dynamics, as for instance the mean sojourn and return times, can be studied in this formalism, allowing an almost complete description of the dynamical properties of the models.

We have introduced the majority rule (MR) as a convenient setting, compared to the null (inertial) majority rule: variables taking values 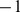 and 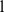 amount to consider that the absence of a regulator has an effect opposite to that observed when the regulator is present. When variables take values 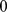 and 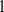, the absence of a regulator is not accounted for in the rule. This has serious consequences: if a node is exclusively subject to inhibitions, there is no configuration for which its value is updated to 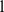, except under the inertial majority rule. The inertial majority rule introduces a self-activation on all the nodes and, for this reason, Li *et al.* as Davidich and Bornholdt, have introduced self-inhibitions on genes that are not negatively regulated otherwise [8], [10].

By thoroughly exploring the properties of simple two-node motifs, we could demonstrate the variety of the behaviours induced by our stochastic extension. Its application to Li *et al.*'s model indicates that it can be used to propose modifications of the model: here, we have shown that to turn the biological state 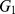 into an absorbing state, one needs to add specific regulatory arcs to the network.

As shortly demonstrated for the cell cycle model, a systematic, efficient method to search for steady (absorbing) states should be relatively easy to implement. Moreover, this method can provide useful indications for model revision in order to ensure that a given state is absorbing. To search for other steady complex behaviours of the revised model, we have generated the corresponding transition diagram. Noticeably, we have verified that 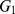 and its mirror states are the sole ergodic states. Future work would focus on a more detailed analysis of the properties of the model such as the nature of the transient dynamics, e.g. providing measures on mean return times.

Extension of the present work also includes the consideration of non-zero thresholds in the majority rule. Importantly, the stochastic extension presented here applies for integer thresholds (considering integer interaction weights); indeed, threshold real values avoid the case of equality in the sum of the regulatory contributions [15]. Note however that, in this case, the probabilistic alternative may be considered as a consequence of uncertainty when gene expression is too close to the theoretical threshold, specially due to local inhomogeneities of protein concentrations.
